# Volatile Compounds, Fatty Acids Constituents, and Antimicrobial Activity of Cultured Spirulina (*Arthrospira fusiformis*) Isolated from Lake Mariout in Egypt

**DOI:** 10.1155/2023/9919814

**Published:** 2023-02-27

**Authors:** Gamal M. Hamad, Nawal Abd El-Baky, Mona Mohamed Sharaf, Amro A. Amara

**Affiliations:** ^1^Food Technology Department, Arid Land Cultivation Research Institute, City of Scientific Research and Technological Applications, New Borg El-Arab City, P.O. Box 21934 Alexandria, Egypt; ^2^Protein Research Department, Genetic Engineering and Biotechnology Research Institute (GEBRI), City of Scientific Research and Technological Applications (SRTA-City), New Borg El-Arab City, P.O. Box 21934 Alexandria, Egypt

## Abstract

In this study, *Arthrospira fusiformis* previously isolated from Lake Mariout (Alexandria, Egypt) was cultivated in the laboratory using a medium for pharmaceutical grade *Arthrospira*, named as Amara and Steinbüchel medium. Hot water extract of the Egyptian *Spirulina* was prepared by autoclaving dried biomass in distilled water at 121°C for 15 min. This algal water extract was analyzed by GC-MS to evaluate its volatile compounds and fatty acids composition. The antimicrobial activity of phycobiliprotein extract from *Arthrospira fusiformis* using phosphate buffer was evaluated against thirteen microbial strains (two Gram-positive bacteria, eight Gram-negative bacteria, one yeast, and two filamentous fungi). The major components of fatty acids in the hot extract of Egyptian *A. fusiformis* were hexadecanoic acid (palmitic acid, 55.19%) and octadecanoic acid (stearic acid, 27.14%). The main constituents of its volatile compounds were acetic acid (43.33%) and oxalic acid (47.98%). The most potent antimicrobial effect of phycobiliprotein extract was obtained against two Gram-negative bacteria *Salmonella typhi* and *Proteus vulgaris*, filamentous fungus *Aspergillus niger*, and the pathogenic yeast *Candida albicans* (all of which showed MIC values of 58.1 *μ*g/ml). *Escherichia coli* and *Salmonella typhimurium* come second in their susceptibility to the phycobiliprotein extract from *Arthrospira fusiformis* and *Serratia marcescens* and *Aspergillus flavus* are the least in susceptibility, with MIC values of 116.2 and 232.5 *μ*g/ml, respectively, while phycobiliprotein extract has no antibacterial effect on methicillin-resistant as well as susceptible *Staphylococcus aureus*, *Pseudomonas aeruginosa*, *Klebsiella pneumoniae*, and *Shigella sonnei*. These findings confirmed the nutritional value of Egyptian *A. fusiformis* isolated from Lake Mariout and suggest the potential use of this strain as an ingredient in the cooking of some foods to increase the level of stearic acid and palmitic acid. Moreover, its effective antibacterial activities against some important and highly resistant to antibiotics bacterial pathogens in addition to its antifungal effects recommend the therapeutic use of its biomass.

## 1. Introduction

The filamentous blue-green microalga *Arthrospira fusiformis* (commercially known as *Spirulina platensis*) was proved to have various nutritional and medicinal properties and was considered a “miracle from the sea” or “superfood” by scientific communities. This edible, microscopic, and alkalophilic cyanobacterium belongs to the microalgae class Cyanophyta [1]. It contains higher amounts of vitamins, protein, minerals, and the like than any other single-cell protein [2, 3]. It has photoautotrophic as well as auxotrophic growth since it has the capability to utilize organic compounds in dark and grow autotrophically in light [4, 5]. Interestingly, there is no need to cook or specially treat *Spirulina* to increase its protein availability, which makes its production simple and preserves its valuable constituents including polyunsaturated fatty acids and vitamins [6].

Due to the nutritional value and pharmaceutical activities of *Arthrospira fusiformis*, it has been used for human and animal nutrition for centuries. This microalga is marketed worldwide in many stores of healthy foods [7–9]. It represents a good supply of bioactive ingredients for the diet including essential amino acids and fatty acids, high protein content (60–70%), pigments of photosynthesis, minerals, carotenoids, and vitamins [10, 11]. Several studies have reported the use of Arthrospira fusiform as a food ingredient (ingredient in pasta and enriched bread) or food additive (added to freeze-dried yogurts) [12–14].

In general, *Arthrospira fusiformis* colonizes unique marine environments that several other microorganisms cannot colonize, such as alkaline saline lakes, which have a pH value of up to 11.0 [1]. It also exists in various habitats such as freshwater, marches, brackish water, seawater, soils, waters of domestic and industrial uses, and thermal springs [15–19]. *Arthrospira fusiformis* exhibits various pharmaceutical activities including antiviral [20, 21], antibacterial [22–24], anti-inflammatory [25–27], antioxidant [24, 28, 29], and anticancer activities [30]. Moreover, it plays a significant role in the treatment of wastewater, the fixation of CO_2_, as well as the production of biofuels, food colors, and methane [31–33].

Five solvents have been commonly used to extract the bioactive ingredients (with antioxidant and antimicrobial activities) of *Arthrospira fusiformis* including ethyl acetate, dichloromethane, methanol, hexane, and petroleum ether [24, 34]. Abedin and Taha [35] tested supernatant, hexane, and methanolic extracts of *Tolypothrix ceytonica*, *Anabaena oryza*, and *Spirulina platensis* for antifungal activity against various phytopathogenic fungi (*Aspergillus niger*, *Fusarium verticillioides*, *Aspergillus flavus*, *Alternaria brassicae*, and *Helminthosporium sp.*) and fungus causing human disease (*Candida albicans*). They found that *Spirulina platensis* extracts have the highest antifungal effect on tested fungi. Kaushik and Chauhan [36] reported that methanol, ethyl acetate, hexane, and dichloromethane extracts of *Spirulina platensis* could inhibit the growth of *Staphylococcus aureus*, *Pseudomonas aeruginosa*, *Escherichia coli*, and *Salmonella typhi*. They showed that methanolic extract has the most potent antibacterial activity, while all extracts have no antibacterial effect on *Klebsiella pneumoniae* [36]. In 2010, Sharaf et al. [20] isolated and authenticated a new Egyptian *Arthrospira fusiformis* strain from the brackish Lake Mariout at the southwest of Alexandria city. The phosphate buffer and hot water extracts of this strain were confirmed to have antiherpetic activity (could inhibit the multiplication of herpesvirus before and during virus infection of host cells) [20, 21].

Najdenski et al. [37] confirmed that ethanol extracts and fatty acids from *Arthrospira fusiformis* have *in vitro* antibacterial activity against *Staphylococcus aureus* and *Streptococcus pyogenes*. Additionally, phycobiliproteins isolated from *Arthrospira fusiformis* were proved to have antimicrobial activity [37]. Ould Bellahcen et al. [38] isolated essential oil by hydrodistillation from *Spirulina platensis* collected from Lake Foum Elouad, Laayoune (South of Morocco) and cultivated in the laboratory using Zarrouk's medium. The essential oil was analyzed by GC/MS and GC/FID. They reported that the major components of essential oils in the Moroccan *Spirulina* were heptadecane (41.7%) and tetradecane (25.3%) [38]. They also reported for the first time in the Moroccan *Spirulina,* the presence of geosmin; a key component of odor in cyanobacteria [38].

A new medium was formulated by Amara and Steinbüchel [39] for pharmaceutical grade *Arthrospira*. The formulated medium was derived from a combination of George's and Zarrouk's media. Amara and Steinbüchel (A-St) medium at concentrations of 1.5–2x (high saline medium) has the ability to inhibit the growth of different forms of cyanobacteria and microalgae including *Chlorella*, whereas only *Arthrospira* could survive in this salinity.

The current work aimed to identify volatile compounds and fatty acids composition extracted by hot water from Egyptian *Arthrospira fusiformis* that was previously isolated from Lake Mariout and authenticated by Sharaf et al. [20]. The hot water extract was analyzed by GC-MS to predict its volatile compounds and fatty acids composition if *Arthrospira* is consumed as an ingredient in cooking or as a hot extract. Additionally, the *in vitro* antimicrobial activities of crude phycobiliprotein sodium phosphate extract of Egyptian *Arthrospira fusiformis* were evaluated against thirteen microbial strains.

## 2. Materials and Methods

### 2.1. Microalga Strain

The extreme alkaliphilic*Arthrospira fusiformis* strain used in this study was isolated from the brackish Lake Mariout at the southwest of Alexandria city (Latitude 31.08011° or 31°4′48″ north, longitude 29.79562° or 29° 47′44″ east, Elevation −26 feet, Open location code 8G3F3QJW +26, GeoNames ID 352723). *Arthrospira fusiformis* was identified via sequencing and analysis of the phycocyanin intergenic spacer region (PC-IGS) in the phycocyanin gene (Accession CBA13040, phycocyanin alpha subunit, partial from *Limnospira fusiformis* LM) by Sharaf et al. [20].

### 2.2. Growth Medium

The composition of Amara and Steinbüchel (A-St) medium 1.5x is as follows: 13.82 g/l NaHCO_3_, 10.71 g/l NaCO_3_, and 0.75 g/l K_2_HPO_4_ (part A); 2.25 g/l NaNO_3_, 0.85 g/l K_2_SO_4_, 1.5 g/l NaCl, 0.22 g/l MgSO_4_.7H_2_O, 0.011 g/l CaCl_2_.2H_2_O, 0.012 g/l FeSO_4_.2H_2_O, and 0.1 g/l EDTA-Na_2_.2H_2_O (part B); 0.02 g/l ferric citrate (part C); and 0.1 g/l peptone, and 0.01 g/l yeast extract (part D) [39]. All parts of this medium except for part D were filter sterilized separately through a syringe filter of 0.22 *μ*m pore size from TPP (St. Louis, MO), while part D was autoclaved. This medium at 1.5x concentration was used for cultivation of the investigated *Arthrospira fusiformis* strain to inhibit the growth of *Chlorella* which was contaminating the seed culture of *Arthrospira.*

### 2.3. Growth Conditions

*Arthrospira fusiformis* was cultivated in Amara and Steinbüchel medium in 20 l bottles. These bottles were aerated and agitated through a sterilized plastic tube using an air pump (150 bubbles/min) [40]. Bottles were incubated at 25°C and light from a florescent lamp/sunlight for 9 days at pH 9.3.

### 2.4. Collection of Microalga

The microalga was collected from 20 l cultivation bottles, washed ten times with double distilled water and dispersed in a mesh bottom frame, and dried at 40°C in an oven until the moisture content reached approximately 13%. The samples were then homogenized using a grinder prior to the extraction method.

### 2.5. Preparation of Water Extract from Microalga

Distilled water (100 ml) was added to 1 g of the dried samples, and the samples were autoclaved at 121°C for 15 min to remove microbes [41, 42]. The autoclaved samples were centrifuged at 2220 ×g for 10 min, and then the supernatant was collected as the water extract solution and lyophilized [43]. We used hot water extraction only to analyze the components consumed by people if they use *Spirulina* as a hot extract or food ingredient in cooking. Algal hot water extract was scanned by GC-MS (Scion GC-456, Netherlands) in Central Laboratory for Scientific Services and Environmental Assessment of SRTA-City (Alexandria, Egypt) to evaluate its volatile compounds and fatty acids composition.

### 2.6. Extraction of Phycobiliproteins

The dried biomass (1 g) was homogenized with 0.1 M sodium phosphate buffer (pH 7) and repeated freezing (at −20°C for 3 h) and thawing (at 4°C for 5 min) were done in dark. Subsequently, the mixture was centrifuged at 10,000*g* and 4°C for 20 min to separate clear supernatant that contains phycobiliproteins [44]. A supernatant sample was run on 12.5% SDS-PAGE. The absorbance of supernatant samples was evaluated at wavelengths 600, 610, 615, 620, 630, 640, 650, and 652 nm (OPTIZEN Scan UV/VIS spectrophotometer, KLab Co., Daejeon, Republic of Korea) for C-phycocyanin.

The concentration of C-phycocyanin was calculated as previously reported by Siegelman and Kycia [45] as follows:

(1) $$\begin{array}{c} C-phycocyanin=\frac{\left(A_{615}\;–\;0.474\times A_{652}\right)}{5.34}\text{,} \end{array}$$

where *A*_615_ is the absorbance measured at 615 nm and *A*_652_ is the absorbance measured at 652 nm.

### 2.7. Test Microorganisms for Antimicrobial Activity

The antimicrobial activity of phycobiliprotein extract from *Arthrospira fusiformis* was tested against thirteen microbial strains. Previously identified methicillin-resistant*Staphylococcus aureus* (MRSA) clinical isolate obtained from the blood of a patient at Almery University Hospital (Alexandria, Egypt) was used in this study [46]. *Candida albicans* ATCC 10231 and *Staphylococcus aureus* ATCC 25923 strains were obtained from Becton Dickinson (France). *Salmonella typhi* ATCC 19430, *Escherichia coli* ATCC 25922, *Salmonella typhimurium* LT2, and *Shigella sonnei* ATCC 25931 were purchased from an American-type culture collection (ATCC, USA). *Aspergillus niger*, *Aspergillus flavus*, *Klebsiella pneumonia*, *Pseudomonas aeruginosa*, *Serratia marcescens*, and *Proteus vulgaris* were collected from Mycology Center of Al-Azhar University (Cairo, Egypt), and Botany and Microbiology Department, Faculty of Science, Al-Azhar University, Assiut Branch (Egypt). A culture aliquot (100 *μ*l) of each strain of bacteria was added to Luria Bertani (LB) broth, incubated overnight at 37°C, and then stored in 20% glycerol at −80°C to be used as seeds stock [47]. Stock cultures of *C. albicans*, *A. niger*, and *A. flavus* were maintained on potato dextrose agar (PDA) overnight at 30°C for *C. albicans* and at 25°C for 5 days for *A. niger* and *A. flavus* [47]. To evaluate antibacterial activity, cation-adjusted Mueller–Hinton (CAMH) broth and Mueller–Hinton agar were used, while antifungal activity was evaluated using potato dextrose broth and potato dextrose agar.

### 2.8. Antimicrobial Activities of Phycobiliprotein Extract from *Arthrospira fusiformis*

Susceptibility screening of test microorganisms to phycobiliprotein extract from *Arthrospira fusiformis* and different antibacterial and antifungal standards was performed using the agar well diffusion technique on Mueller-Hinton agar and Potato dextrose agar for bacteria and fungi, respectively [47]. Plates were overlaid with 100 *μ*l of an overnight culture of each bacterial pathogen at a cell density of 10^6^ CFU/ml, and 100 *μ*l of an overnight culture of *C. albicans* at a cell density of 10^4^ cells/ml based on McFarland turbidity standard [47, 48]. Potato dextrose agar plates were overlaid with 1 ml fungal suspension (6 mm disc of the fungal growth suspended in sterile water) of 5 days old culture of *A. niger* and *A. flavus*. Then, wells were cut in the agar media by using a sterile 6 mm cork borer and filled with 100 *μ*l of phycobiliprotein extract at a concentration of 0.93 mg/ml. Fusidic acid standard disc obtained from Mast Diagnostics (Merseyside, UK), chloramphenicol from Bioshop (Ontario, Canada), or amphotericin-B purchased from HyClone (Logan, Utah, USA) were used as a positive control, while sterile water was used as a negative control. The culture plates were kept at 4°C for 2 h to allow proper diffusion of tested antimicrobials through the inoculated media before being incubated at 30°C and 37°C for 24 h in the case of *C. albicans* and bacterial cultures, respectively, and for 5 days at 25°C in case of fungal cultures. The presence of the inhibition zones was examined and recorded in (mm) of three replicates.

### 2.9. Broth Microdilution Susceptibility Assay

The minimum inhibitory concentrations (MICs) of phycobiliprotein extract from *Arthrospira fusiformis* against test pathogens were determined by broth microdilution technique [49]. Two 96-well microtiter plates (Greiner, Frickenhausen, Germany) were inoculated with test microorganisms that were susceptible to the phycobiliprotein extract in the agar well diffusion assay, and then 100 *μ*l of CAMH broth or potato dextrose broth containing phycobiliprotein extract in two-fold serial dilutions were added. The concentrations of phycobiliprotein extract ranged from 58.12 *μ*g/ml to 0.93 mg/ml. The plate inoculated with bacteria and *C. albicans* was incubated at 30°C for 24 h, whereas the plate inoculated with fungi was incubated at 25°C for 5 days. The MICs were determined by measuring the absorbance at 600 nm to test bacterial pathogens and *C. albicans* and calculating fungal sporulation using a hemocytometer. All MIC determinations were performed in duplicate. The MIC was defined as the lowest concentration at which growth was completely inhibited. Bacteria in CAMH broth and fungi in potato dextrose broth were used as control.

### 2.10. Statistical Analysis

The susceptibility assay of test microorganisms to phycobiliprotein extract from *Arthrospira fusiformis* was carried out in triplicate and the obtained results were demonstrated as mean ± SD of triplicate. Data analysis was done by using Student's *t*-test and McNemar's test. A *P* value of less than 0.05 was regarded as statistically significant.

## 3. Results

### 3.1. GC-MS Analysis of Algal Hot Water Extract

The percentage composition of the volatile compounds and fatty acids identified in the hot water extract of *Arthrospira fusiformis* isolated from Lake Mariout and cultivated in Amara and Steinbüchel medium is presented in Table 1. A total of two fatty acids, one fatty acid methyl ester, and three volatile compounds were identified. The major components of *A. fusiformis* fatty acids were hexadecanoic acid (palmitic acid, 55.19%) and octadecanoic acid (stearic acid, 27.14%). The main constituents of its volatile compounds were acetic acid (43.33%) and oxalic acid (47.98%). GC-MS chromatograms of fatty acids and volatile compounds composition of algal hot water extract are presented in Figures S1 and S2. GC-MS chromatogram of algal hot water extract revealed the presence of a secondary metabolite or bioactive (2H-Pyran, tetrahydro-2-(12-pentadecynyloxy), and monoterpenoid (3,7-dimethyl-1,6-octadiene).

### 3.2. Extraction of Phycobiliproteins

**Figure 1:**
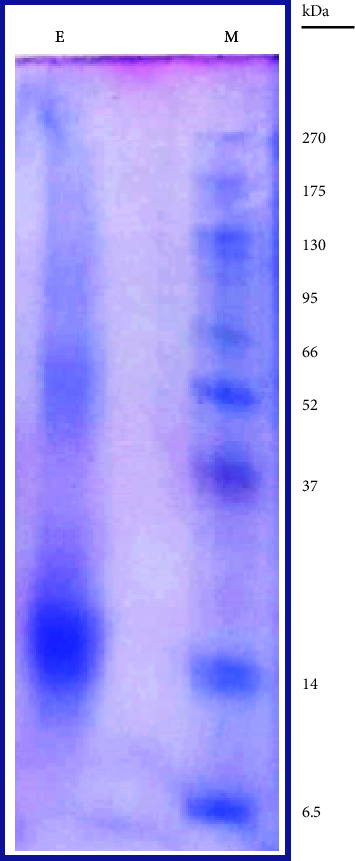
12.5% SDS-PAGE analysis of crude phycobiliproteins extract. M: protein molecular weight marker. E: phycobiliproteins extract containing a dense band of more than 14 kDa corresponding to phycocyanin subunits.

**Figure 2:**
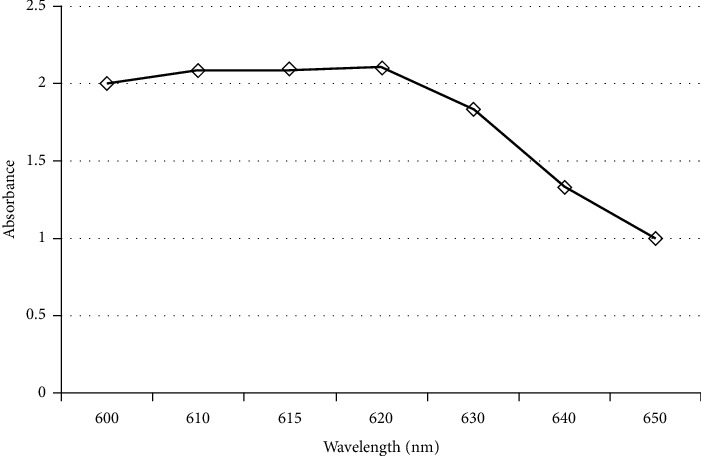
Visible spectrum (at wavelengths 600–650 nm) of C-phycocyanin in phycobiliproteins extract.

The phycobiliproteins extraction using 0.1 M sodium phosphate buffer (pH 7) yielded 0.93 ± 0.1 mg/ml phycobiliproteins. SDS-PAGE analysis of phycobiliproteins extract is presented in Figure 1. The visible spectrum (at wavelengths 600–650 nm) of C-phycocyanin in phycobiliproteins extract is illustrated in Figure 2. The concentration of C-phycocyanin (mg/ml) calculated by the equation of Siegelman and Kycia [45] was 0.308. The purity of C-phycocyanin in phycobiliproteins extract calculated by dividing *A*_615_ (=2.094) by *A*_280_ (=2.55) was 0.82.

### 3.3. Antimicrobial Activities of Phycobiliprotein Extract from *Arthrospira fusiformis*

The test microorganisms differed in their susceptibility to phycobiliprotein extract from *Arthrospira fusiformis*. The Gram-positive bacteria MRSA and *S. aureus* in addition to the Gram-negative bacteria *P. aeruginosa*, *K. pneumonia*, and *S. sonnei* were resistant (no inhibition zones) to the phycobiliprotein extract. The most potent antimicrobial effect of phycobiliprotein extract was obtained against two Gram-negative bacteria *Salmonella typhi* and *Proteus vulgaris*, filamentous fungus *Aspergillus niger*, and the yeast *Candida albicans* (with the largest inhibition zone of 20 mm) as recorded in Table 2. Test microorganisms that showed susceptibility (inhibition zones) to phycobiliprotein extract from *Arthrospira fusiformis* are demonstrated in Figure S3.

### 3.4. Broth Microdilution Susceptibility Assay

Results of broth microdilution susceptibility assay revealed that *S. typhi*, *P. vulgaris*, *C. albicans*, and *A. niger* were the most susceptible test organisms with minimal inhibition concentration of 58.1 *μ*g/ml, while the Gram-negative bacterium *S. marcescens* and the filamentous fungus *A. flavus* were the least susceptible ones (MIC of 232.5 *μ*g/ml) (Table 3).

## 4. Discussion

The prokaryotic microalga *Arthrospira fusiformis* was isolated from soda lakes, seawater, as well as freshwater [50]. Commercial production of this microalga started about three decades ago and currently, *Arthrospira fusiformis* or *Spirulina* is widely consumed in vitamin supplements, nutraceuticals, and pharmaceuticals besides other applications in food dyes, aquaculture, and fish food [8, 30, 38, 51]. *Arthrospira fusiformis* provides its consumer with high protein content (percent composition of 60–70 by wet weight), which is considered as a complete protein (comprising eight essential amino acids such as isoleucine, leucine, in addition to valine, nevertheless with lower cysteine, methionine, and lysine content compared to standard proteins and ten nonessential amino acids) [10, 11, 52]. It also provides carbohydrates, polyunsaturated fatty acids, vitamins, essential minerals, bioactive peptides, and pigments [53, 54]. *Arthrospira fusiformis* contains predominantly two phycobiliproteins (C-phycocyanin and allophycocyanin) [55]. Both phycobiliproteins have various applications as potential pharmaceuticals in oxidative stress-induced diseases and promising phycocyanin-based anticancer treatments [56, 57]. Furthermore, phycobiliproteins isolated from *Arthrospira fusiformis* were proved to have antimicrobial activity [37]. C-phycocyanin is a blue natural pigment commonly used as a natural colorant for food additive purposes and has also anti-inflammatory activities [58, 59]. Even fatty acids from *Arthrospira fusiformis* were found to have *in vitro* antibacterial activity against *Staphylococcus aureus* and *Streptococcus pyogenes* [37]. The effective health benefits of *Arthrospira fusiformis* have been extensively detailed in many scientific reports [10, 20, 21, 23, 26–30, 60–62].

In 2010, Sharaf et al. [20] isolated a new *Arthrospira fusiformis* strain from the brackish Lake Mariout at the southwest of Alexandria city in Egypt. The strain was proved to have unique tolerance for salinity, and its phosphate buffer and hot water extracts could directly inactivate herpes simplex viral particles before infection of host cells. They recommended applying *Spirulina* crude extracts as a treatment for recurrent herpetic infection [20].

In this study, we identified volatile compounds and fatty acids composition extracted by hot water from *Arthrospira fusiformis* isolated by Sharaf et al. [20] and tested the antimicrobial activities of its phycobiliprotein extract using sodium phosphate buffer. The obtained results revealed that two fatty acids, one fatty acid methyl ester, and three volatile compounds were identified in the hot water extract of *Arthrospira fusiformis*. The major components of its fatty acids were palmitic acid (55.19%), and stearic acid (27.14%). Our results support and agree with a previous report by Abd El-Hameed et al. [63], which demonstrated that adding *Spirulina platensis* (*Arthrospira fusiformis*) to raw and cooked spaghetti leads to an increase of five fatty acids including palmitic acid, and stearic acid. They also observed that the fatty acid profile of spaghetti prepared with the incorporation of *Spirulina platensis* has a higher resistance to the thermal treatment applied during the cooking [63]. The main constituents of volatile compounds in hot extract were acetic acid (43.33%), and oxalic acid (47.98%). GC-MS chromatogram of algal hot water extract also revealed the presence of a secondary metabolite, 2H-Pyran, tetrahydro-2-(12-pentadecynyloxy, with a percentage of 3.7. This secondary metabolite has potential application as an inhibitor to non-small cell lung cancer as well as antibacterial and antioxidant activities [64, 65].

In the present study, results obtained from antimicrobial activities assays of the phycobiliprotein extract of *Arthrospira fusiformis* revealed that eight of the tested bacterial and fungal pathogens were susceptible to the phycobiliprotein extract but the most potent antimicrobial effects were observed against *Salmonella typhi*, *Proteus vulgaris*, *Aspergillus niger*, and *Candida albicans*. No antibacterial effect was found on methicillin-resistant as well as susceptible *Staphylococcus aureus*, *Pseudomonas aeruginosa*, *Klebsiella pneumoniae*, and *Shigella sonnei*. These results agree in part with previous reports by Murugan [66], and Zamani et al. [67], which showed that C-phycocyanin of *Arthrospira fusiformis* has antifungal activities against *Aspergillus niger*, *Aspergillus flavus*, and *Candida albicans.* These results also are in agreement with previous studies by Sun et al. [22], Sadeghi et al. [23], and Abdel-Moneim et al. [24], which confirmed antibacterial activities of *Spirulina platensis* extracts, proteins, and peptides against selected pathogenic bacteria. However, our data disagree with the previous study by Sarada et al. [68], which demonstrated that C-phycocyanin from *Spirulina platensis* (Nordstedt) Geitler could inhibit the growth of *Klebsiella pneumoniae*, *Pseudomonas aeruginosa, and Staphylococcus aureus*.

The results obtained from this work suggest the potential use of biomass of *A. fusiformis* isolated from Lake Mariout as a food additive. Our findings combined with the results obtained by Sharaf et al. [20, 21] also recommend that *A. fusiformis* isolated from Lake Mariout is a potential antimicrobial against some bacterial and fungal pathogens and antiviral therapeutic.

## 5. Conclusion

There is a growing interest in the use of microalga *Arthrospira fusiformis* not only for food applications but also as safe pharmaceutical products with antimicrobial and antioxidant activities as well as corrective properties against tumor growth, anemia, and malnutrition. Therefore, we cultivated a previously isolated Egyptian *Arthrospira fusiformis* in the laboratory and analyzed its volatile compounds and fatty acids composition in the hot extract. We used hot water extraction only to analyze the components (volatile compounds and fatty acids) consumed by people if they use *Spirulina* as a hot extract or food ingredient in cooking. The antimicrobial activity of its phycobiliprotein extract was also evaluated against thirteen pathogens, eight of these pathogens were susceptible to the phycobiliprotein extract. The major fatty acids found were palmitic acid and stearic acid, which suggests the promising application of Egyptian *A. fusiformis* biomass as an ingredient in the cooking of some foods such as pasta or bread to increase the level of the two most commonly consumed saturated fatty acids; stearic acid and palmitic acid. Overall, our findings revealed that *A. fusiformis* isolated from Lake Mariout has nutritional as well as effective antimicrobial properties and suggest the potential therapeutic use of its biomass.

## Figures and Tables

**Table 1:** The percentage composition of the volatile compounds and fatty acids identified in hot water extract of *Arthrospira fusiformis* isolated from lake Mariout.

No	Compounds	Type	Percentage (%)
1	Methyl tetradecanoate	Fatty acid methyl ester	10.31
2	9-Octadecenoic acid methyl ester	Fatty acid methyl ester	0
3	Hexadecanoic acid, methyl ester (palmitic acid)	Fatty acid	55.19
4	Phthalic acid, cyclobutyl decyl ester	Natural phthalate esters	2.77
5	2H-pyran, tetrahydro-2-(12-pentadecynyloxy)	Secondary metabolites (bioactive)	3.7
6	9,12-octadecadienoic acid, methyl ester (linoleic acid)	Fatty acid	0
7	3,7-Dimethyl-1,6-octadiene	Monoterpenoids found in the essential oils	0.89
8	Octadecanoic acid, methyl ester (stearic acid)	Fatty acid	27.14
9	Acetic acid	Volatile compound	43.33
10	Oxalic acid, isobutyl pentyl ester	Volatile compound	47.98
11	Benzo [1,3] dioxole-5-carboxylic acid	Volatile compound	0
12	Isoamyl nitrite	Volatile compound	8.69

**Table 2:** Susceptibility of test microorganisms to phycobiliprotein extract from *Arthrospira fusiformis*.

Test strains	Mean diameter of inhibition zone^a^ (mean ± SD)			
	Phycobiliprotein extract	C	AMP	FC
MRSA	R	R	NT	22 ± 1.63
*S. aureus*	R	14.66 ± 1.24	NT	NT
*E. coli*	9 ± 0.81	14 ± 1.63	NT	NT
*S. typhi*	19.66 ± 0.94	18.33 ± 0.47	NT	NT
*S. typhimurium*	10.33 ± 0.47	22 ± 1.63	NT	NT
*P. aeruginosa*	R	8 ± 0.81	NT	NT
*K. pneumonia*	R	R	NT	NT
*S. sonnei*	R	12 ± 0.81	NT	NT
*P. vulgaris*	14 ± 0.81	24.33 ± 0.47	NT	NT
*S. marcescens*	5.33 ± 0.47	14.66 ± 0.47	NT	NT
*C. albicans*	20 ± 1.63	NT	13 ± 0.81	NT
*A. niger*	12.66 ± 0.47	NT	14.33 ± 0.47	NT
*A. flavus*	7.66 ± 0.47	NT	7.66 ± 0.47	NT

^a^Mean of three assays; C-chloramphenicol and FC-fusidic acid antibacterial standards at concentrations of 50 and 10 *μ*g/ml, respectively; AMP-amphotericin-B antifungal standard at a concentration of 100 *μ*g/ml; R-resistant (no inhibition zone); NT-not tested.

**Table 3:** MIC values of phycobiliprotein extract from *Arthrospira fusiformis* against tested susceptible pathogens.

Test strains	MIC values (*μ*g/ml)
*E. coli*	116.2
*S. typhi*	58.1
*S. typhimurium*	116.2
*P. vulgaris*	58.1
*S. marcescens*	232.5
*C. albicans*	58.1
*A. niger*	58.1
*A. flavus*	232.5

## Data Availability

Data are contained within the article and supplementary materials.

## Supplementary Materials

Supplementary Materials Figure S1: GC-MS chromatogram of fatty acids composition of algal hot water extract. Figure S2: GC-MS chromatogram of volatile compounds composition of algal hot water extract. Figure S3: Test microorganisms that showed susceptibility to phycobiliprotein extract from *Arthrospira fusiformis.*
