# Supplementary material for: Volatile Compounds, Fatty Acids Constituents, and Antimicrobial Activity of Cultured Spirulina (Arthrospira fusiformis) Isolated from Lake Mariout in Egypt
Source: ScientificWorldJournal. 2023 Feb 27;2023:9919814. doi: 10.1155/2023/9919814 (PMC9988382; doi:10.1155/2023/9919814)
Supplement: Supplementary Materials — Figure S1: GC-MS chromatogram of fatty acids composition of algal hot water extract. Figure S2: GC-MS chromatogram of volatile compounds composition of algal hot water extract. Figure S3: Test microorganisms that showed susceptibility to phycobiliprotein extract from Arthrospira fusiformis. [file 9919814.f1.docx]

**Volatile compounds, Fatty acids constituents and antimicrobial activity of cultured *Spirulina* (*Arthrospira fusiformis*) isolated from Lake Mariout in Egypt**

Gamal M. Hamad^1, #^, Nawal Abd El-Baky^2, #, *^, Mona Mohamed Sharaf^2^, Amro A. Amara^2, *^

*^1^Food Technology Department, Arid Land Cultivation Research Institute, City of Scientific Research and Technological Applications, New Borg AL Arab, Alexandria, Egypt*

^2^*Protein Research Department, Genetic Engineering and Biotechnology Research Institute (GEBRI), City of Scientific Research and Technological Applications (SRTA-City), New Borg El-Arab City, P.O. Box 21934 Alexandria, Egypt*

^#^These authors contributed equally to this work.

Correspondence:

Nawal Abd El-Baky, Protein Research Department, Genetic Engineering and Biotechnology Research Institute, City of Scientific Research and Technological Applications, New Borg El-Arab City, Alexandria, Egypt

nelbaky@srtacity.sci.eg; Tel.: +20-3459-3422; Fax: +20-3459-3407

Amro A. Amara, Protein Research Department, Genetic Engineering and Biotechnology Research Institute, City of Scientific Research and Technological Applications, New Borg El-Arab City, Alexandria, Egypt

*aamara@srtacity.sci.eg; amroamara@web.de;* Tel.: +20-3459-3422; Fax: +20-3459-3407

**Running title:** Chemical composition and antimicrobial activity of *Arthrospira fusiformis* from Egypt.


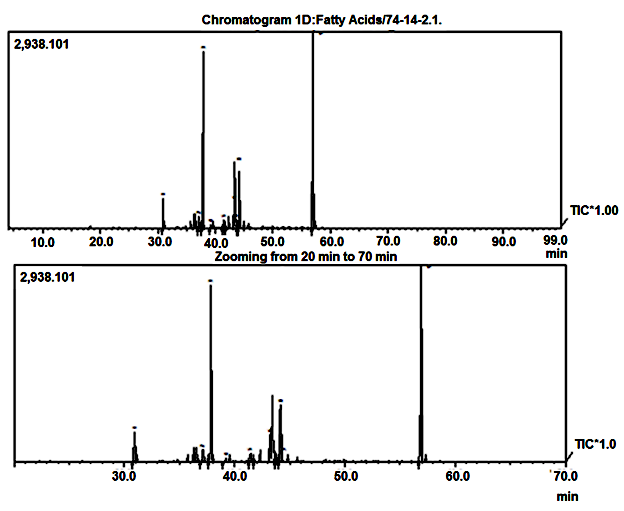

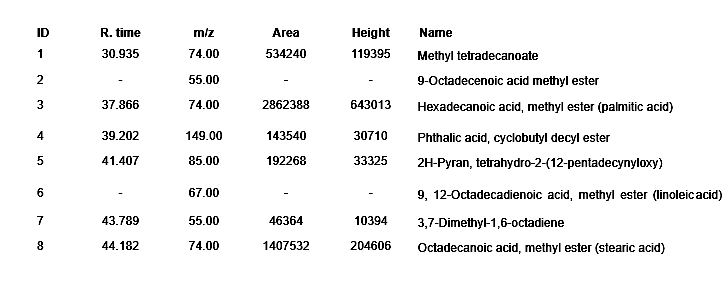


**Figure S1.** GC-MS chromatogram of fatty acids composition of algal hot water extract. The m/z value represents mass divided by charge number of ions. The horizontal axis in a mass spectrum is expressed in m/z units. As z is almost permanently 1 with GC-MS, the m/z value is often considered to be the mass.


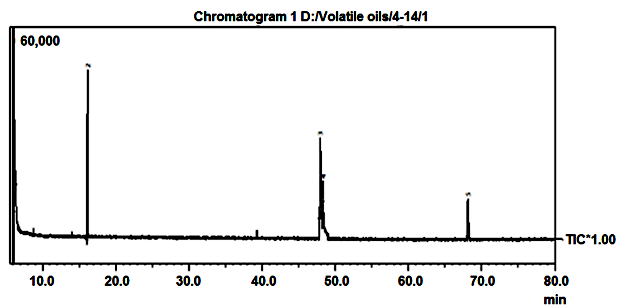

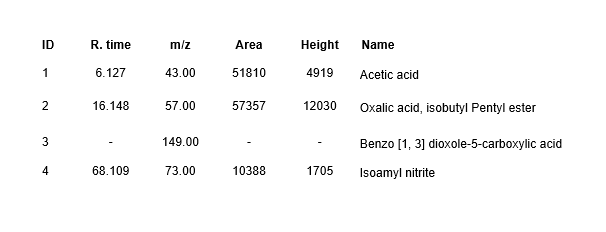


**Figure S2.** GC-MS chromatogram of volatile compounds composition of algal hot water extract. The m/z value represents mass divided by charge number of ions. The horizontal axis in a mass spectrum is expressed in m/z units. As z is almost permanently 1 with GC-MS, the m/z value is often considered to be the mass.


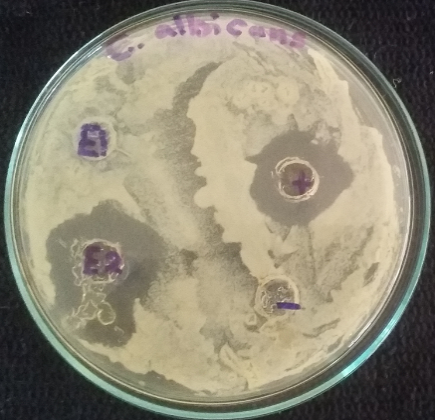

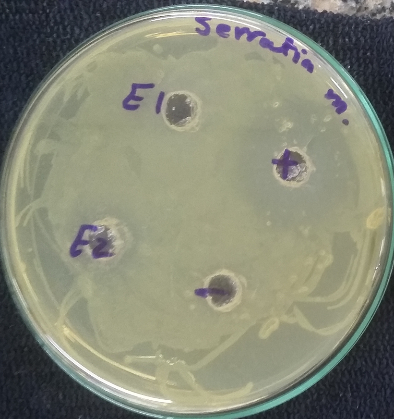

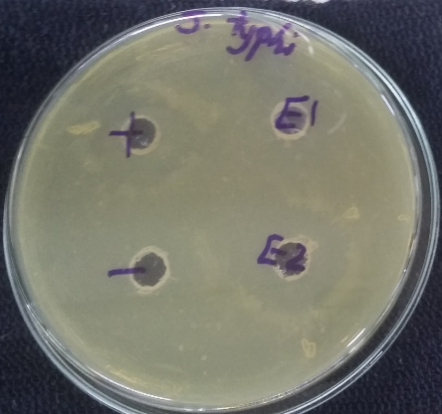

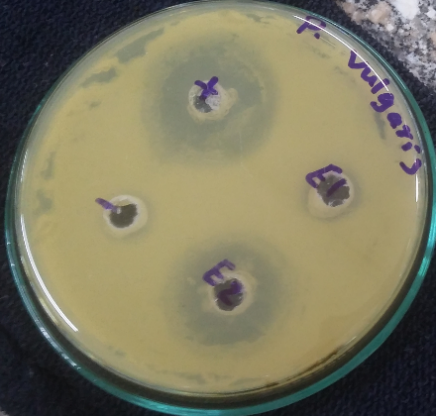

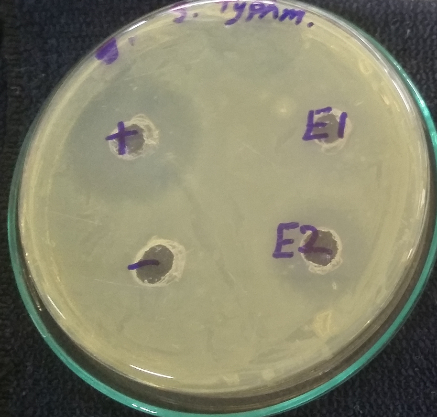

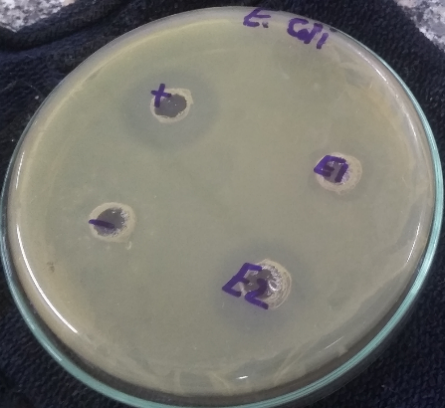

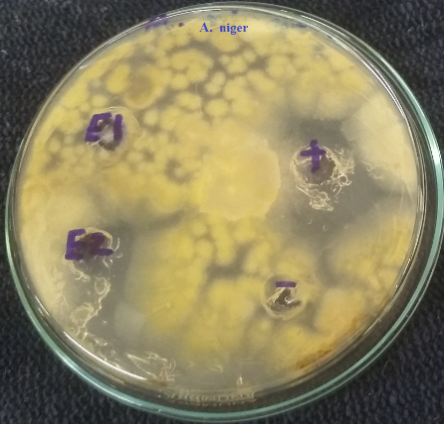

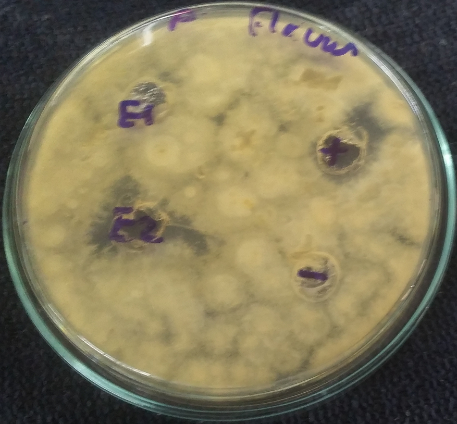


**Figure S3.** Test microorganisms that showed susceptibility to phycobiliprotein extract from *Arthrospira fusiformis.* + represent positive control (chloramphenicol or amphotericin-B). – represent negative control (sterile water). E1 represent algal hot water extract. E2 represent phycobiliprotein extract.
